# Metabolite analysis of *Aspergillus fumigatus* conidial supernatant and its pro-inflammatory activity *in vitro* and *in vivo*

**DOI:** 10.3389/fimmu.2025.1730825

**Published:** 2026-01-27

**Authors:** Qiujie Li, Shan Li, Yuting Kang, Jian Xue, Pengtao Wang, Wei Jia

**Affiliations:** 1 First Clinical Medical College, Ningxia Medical University, Yinchuan, China; 2 Senior Department of Cardiology, The Sixth Medical Center of Chinese PLA General Hospital, Beijing, China; 3 Ningxia Key Laboratory of Clinical and Pathogenic Microbiology, Institute of Medical Sciences, General Hospital of Ningxia Medical University, Yinchuan, China; 4 Center of Medical Laboratory, General Hospital of Ningxia Medical University, Yinchuan, China

**Keywords:** *A. fumigatus*, conidia, germination, inflammatory response, metabolite

## Abstract

**Introduction:**

*Aspergillus fumigatus* (*A. fumigatus*) conidia have been reported to induce inflammatory response in macrophages, resulting in lung damage. However, the role of secondary metabolites secreted by conidia during the infection process remains unclear. Our objective is to investigate the metabolic changes produced by conidia at different developmental stages and to assess the effects of the conidial supernatant on the inflammatory response of macrophages.

**Methods:**

We employed optical microscopy, electron microscopy, and nuclear division staining to identify the morphological characteristics of the *Aspergillus fumigatus* strain Af293 conidia at various developmental stages. Metabolomic analysis of the supernatant from conidial pre-germination (Af293-4h) and post-germination (Af293-12h) was performed using Liquid Chromatography-Mass Spectrometry. Conidial supernatant was utilized to stimulate mouse alveolar macrophages (MH-S) cells, and the expression of inflammatory factors was quantified using ELISA and RT-qPCR. Western blotting was conducted to detect the levels of key proteins involved in the inflammatory pathway. Furthermore, mice were administered an intranasal instillation of the supernatant to construct the pneumonia model, and lung pathology was evaluated through hematoxylin-eosin (HE) staining, while the levels of inflammatory factors in bronchoalveolar lavage fluid were assessed using ELISA and RT-qPCR.

**Results:**

Non-targeted metabolomics analyses reveal an increased secretion of organic acids and their derivatives, lipids and lipid-like molecules, phenolic compounds, phenylpropanoids, polyketides, as well as alkaloids and their derivatives following conidial germination. Compared to Af293-4h supernatant, Af293-12h supernatant induce a significantly stronger inflammatory response in MH-S cells, characterized by the increased expression of inflammatory factors, including IL-1β, TNF-α, CCL/CXCL and MMPs, via the activation of JAK/STAT/AKT and MAPK signaling pathways. Nasal exposure of conidial supernatant in mice can induce lung inflammation, resulting in lung damage and an elevated proportion of inflammatory cells, as well as increased levels of the inflammatory factors such as TNF-α, IL-1β, and IL-6.

**Conclusion:**

Our research indicates significant differences in the metabolites of *A. fumigatus* conidial supernatant between the pre-germination and post-germination stages. The conidial supernatant can induce a pronounced inflammatory response in macrophages, mediated by the activation of the JAK/STAT/MAPK pathways. Long-term exposure to spore supernatant in mice can result in pneumonia and tissue damage.

## 1 Introduction

*Aspergillus fumigatus* (*A. fumigatus*) is a filamentous, airborne saprophytic fungus that contributes to elevated rates of pulmonary infections and mortality in humans, thus imposing a substantial burden on global health (1). *A. fumigatus* typically exists in the air as conidia, which are generally cleared by the mucociliary system and resident macrophages in healthy individuals (2–4). However, in immunocompromised patients, the lung tissue’s ability to clear conidia is compromised, leading to increased adhesion and germination of *A. fumigatus*, which can result in invasive aspergillosis (IA) (5–7). Research has demonstrated that *A. fumigatus* conidia can induce inflammatory response in pulmonary epithelial cells and macrophages, causing a robust inflammatory reaction in immunocompetent mice and resulting in pathological damage to the lungs (8–10). Moreover, conidia possess various secretory substances, including multiple proteases, allergens, and toxins, and other secondary metabolites, to evade macrophage phagocytosis (11, 12). One such mycotoxin, gliotoxin, has been shown to disrupt the pulmonary epithelial barrier and promote immune evasion (13–15). It has been verified that conidial supernatant can damage the bronchial epithelial monolayer, exacerbating the clinical symptoms of cystic fibrosis (14). These findings indicate that, in addition to conidia, its secondary metabolites also exert a detrimental effect on lung tissue, warranting further investigation.

Macrophages are a crucial component of the innate immune system, playing a pivotal role in pathogen recognition, inflammation regulation, and tissue homeostasis. Traditionally, it has been believed that adult macrophages primarily originate from bone marrow monocytes; however, recent lineage-tracing and single-cell studies have demonstrated that many tissue-resident macrophages (TRMs) have an embryonic origin, deriving from yolk sac or fetal liver progenitors, and can be maintained long-term through local self-renewal (16, 17). Alveolar macrophages are representative of embryonically derived TRMs and primarily depend on self-renewal under steady-state conditions. During instances of infection or tissue injury, bone marrow-derived monocytes supplement and differentiate into inflammatory macrophages. Upon encountering fungal invasions, macrophages can recognize fungi, activate host immunity, and eliminate condia to inhibit fungal proliferation (18). Recent research has established that the dectin-1 receptor on alveolar macrophages can recognize β-glucans on the surface of fungal cells (19), thereby activating the MAPK signaling pathway to initiate the innate immune response of these macrophages (20). Furthermore, both mouse and human alveolar macrophages can phagocytose *A. fumigatus* conidia through mechanisms involving actin polymerization and phosphatidylinositol 3-kinase activation (21). It has also been found that the melanin present on the surface of conidia can modulate glucose and glutamine metabolism in macrophages, promoting cellular inflammation (22, 23). Research show that after infection with *A. fumigatus*, various pro-inflammatory factors are elevated in macrophage cell line RAW264.7, including TNF-α, IL-1β, IL-1α, CXCL-1, CXCL-2, and GM-CSF, leading to an inflammatory response (8, 24). *A. fumigatus* conidia induces higher levels of cytokines TNF-α, IL-6, IL-1β, and IL-18 in bone marrow-derived macrophages (25). Aspergillus fumigatus can induce pulmonary inflammatory responses in mice by promoting the expression of various inflammatory factors such as CXCL-2, IL-1α, CXCL-1, GM-CSF, IL-1β, IL-6, and MIP-1α (8). However, the role of metabolites produced by *A. fumigatus* during the infection process in macrophages remains unclear and requires further elucidation.

In recent years, studies on the secondary metabolism of *A. fumigatus* have demonstrated that the metabolic processes of filamentous fungi are influenced by various conditions, including nitrogen sources, carbon sources, pH levels, temperature, and light (26). This metabolism is regulated at multiple levels, including transcriptional, epigenetic, and post-translational (27–29). As a result, the metabolic changes that occur during the germination of conidia are complex, leading to a diverse array of secondary metabolites. These metabolites play crucial roles in a variety of biological processes, such as heat resistance, cell wall composition and maintenance, fulfillment of nutritional requirements, interactions with the host immune system, and responses to stress (1). Notable secondary metabolites include gliotoxin, fumonisin, verruculogen, restrictocin, helvolic acid, and proteases, each playing distinct ecological roles in fungal defense, dissemination, and virulence, particularly during infections, thereby impacting host cells (30). The elastase and collagenase secreted by *A. fumigatus* can degrade alveolar tissue and disrupt the integrity of the epithelial barrier (31). Gliotoxin has been extensively studied for its cytotoxic and immunosuppressive properties, which have been documented in previous reports (32–34). However, these toxins are secreted only after the conidia germinate into hyphae (approximately 24 hours) (35–37), while the metabolite components of the early stages of conidia (before 12 hours) and their effects on host cells remain poorly understood. Therefore, we analyzed the differentially expressed metabolites secreted by *A. fumigatus* during two early growth stages (conidia and hyphae) using mass spectrometry and preliminarily explored the inflammatory response of these metabolites on macrophages. Furthermore, we established an *in vivo* model by performing intranasal instillation of mice with conidial supernatant to assess its effects on inflammation.

## 2 Materials and methods

### 2.1 Maintenance, storage, and culture condition of *A. fumigatus* strain Af293

In this study, the *A. fumigatus* strain Af293 (ATCC MYA-4609) was generously provided by Prof. Liu Wei from Peking University First Hospital. The fungal isolate was cultured on Sabouraud dextrose agar (SDA) medium at 37°C for a period of 5 to 7 days. Subsequently, the culture was treated with a washed solution of 0.05% Tween 80, and the conidia of Af293 were gently scraped off using a disposable pipette to obtain a conidia suspension. This suspension was then stored at -80°C in the Medical Laboratory Center of the General Hospital of Ningxia Medical University for long-term preservation for future research applications.

### 2.2 Determination of Af293 conidial germination rate

The prepared conidial suspensions were inoculated into seven 50 mL centrifuge tubes, each containing 15 mL of Czapek medium, achieving a final concentration of 1 × 10^5^ conidia/mL. The conidial concentration was quantified by diluting the prepared conidial suspension in a gradient from 1:10 to 1:1000 as necessary. The conidia were counted using a Neubauer counting chamber under an optical microscope. The conidial concentration was calculated using the formula (average count × 10^4^ × dilution factor), ensuring a final concentration of 1 × 10^5^ conidia/mL. The tubes were incubated in a shaking incubator at 37°C and 200 rpm for durations of 0, 2, 4, 6, 8, 10, and 12 hours. At each time point, a small sample was placed on a microscope slide, covered with a coverslip, and examined under a standard optical microscope to observe the germination of 100–200 conidia. Germination was defined as a germ tube length that is greater than or equal to the conidia radius. The conidia germination rate was calculated using the formula: Germination rate (%) = (a/A) × 100, where ‘a’ represents the number of germinated conidia (units) and ‘A’ denotes the total number of conidia observed (units).

### 2.3 Observation of Af293 conidia germination morphology

For optical microscopy, samples collected from the seven time points described in Section 2.2 were placed on slides, covered with coverslips, and observed under a standard optical microscope at 40 × magnification, with photographic documentation accompanying the observations. For scanning electron microscopy (SEM), samples from the seven time points outlined in Section 2 were washed 1–2 times with phosphate-buffered saline (PBS), fixed in 3% glutaraldehyde, and subsequently rinsed three times with ultrapure water for 10 minutes each. The samples were then fixed with 1% osmium tetroxide for 1–2 hours, followed by three additional rinses with ultrapure water (10 minutes each), and dehydrated stepwise with ethanol at concentrations of 30%, 50%, 70%, 90%, and 100% (15 minutes per step at 100% concentration). A drop of the sample was placed on a coverslip and dried in a critical point dryer. Finally, the coverslip was attached to the sample stage using conductive adhesive and placed in an ion sputter coater for gold coating. Image acquisition was performed using a JEOL JSM-IT700HR scanning electron microscope.

### 2.4 Cell nuclear division staining of Af293 conidia

Samples from the seven time points in Step 2.2 were washed 1–2 times with PBS, followed by fixation at room temperature for 2 hours using a 2.5% glutaraldehyde solution. After fixation, the samples were washed again 1–2 times with PBS and stained with a DAPI working solution at room temperature for 20 minutes. Subsequently, the samples were washed once more with PBS, prepared as slides, mounted, and observed under a fluorescence microscope for photographic documentation.

### 2.5 Maintenance of murine alveolar macrophages (MH-S) cell lines

In this study, MH-S were cultured in RPMI 1640 growth medium supplemented with 10% fetal bovine serum (FBS) and 1% penicillin-streptomycin. They were maintained under controlled conditions of 5% CO_2_ at 37°C. MH-S cells were periodically subcultured and used during the exponential growth phase for the experiments.

### 2.6 Treatment of MH-S cells with supernatant from Af293 conidia

Af293 conidia were cultured in SDA medium at a concentration of 1 × 10^5^ conidia/mL for durations of 4 hours and 12 hours, respectively. The supernatant were collected and passed through a sterile cell sieve, followed by sterile filtration using a 0.22 µm syringe filter (Merck Millipore) to eliminate all conidia and hyphae, achieving a final concentration of 100%. Subsequently, cells were stimulated with the supernatant from both 4-hour and 12-hour cultures to obtain a 1% concentration of supernatant in complete medium.

### 2.7 Conidial supernatant induced lung inflammation in the mouse model

The animal experiments were approved by the Institutional Animal Ethics Research Board of the General Hospital of Ningxia Medical University (KYLL-2024-0344). A total of 10 male BALB/c mice, aged 4 to 6 weeks and weighing between 18 and 20 grams, were obtained from Cloud-Clone Corp. The mice were housed in ventilated racks within the animal facility, with ad libitum access to food and water, and maintained on a 12-hour light/dark cycle. After a week of acclimatization, the mice were randomly divided into two equal groups: the Control (CTRL) group and the Conidial Supernatant-induced Lung Inflammation (CSLI) group. Mice in the CSLI group received a single intranasal instillation of 1% conidial supernatant (CS) of Af293-12h, diluted with phosphate-buffered saline (PBS), twice daily for one month, while the control group received PBS twice daily for the same duration.

### 2.8 Histopathological examination

To collect bronchoalveolar lavage fluid (BALF) samples, the mice were first anesthetized, and their tracheas were surgically exposed. Subsequently, the left lungs were washed twice with 2 ml of cold phosphate-buffered saline (PBS). Following this, BALF was centrifuged at 12,000×g for 10 minutes at 4°C, and the supernatant was collected for cytokine analysis using ELISA kits. The pellet was immediately processed for cell sorting, with white blood cells classified via Wright-Giemsa staining kit (G1079, Servicebio, China). For the right lungs, tissue samples were collected, fixed in 4% paraformaldehyde, embedded in paraffin, sectioned to a thickness of 5 µm, and stained using the HE Staining Kit (G1120, Solarbio, China).

### 2.9 RNA extraction and cytokine gene expression

Total RNA was extracted from conidia, MH-S lung macrophages and lung tissue following the manufacturer’s instructions using TRIzol reagent (Thermo Scientific, Rockford, IL, USA). The total RNA was reverse-transcribed into cDNA using a first-strand cDNA synthesis kit (TaKaRa, Shiga, Japan), in accordance with the manufacturer’s guidelines. The resulting cDNA was amplified with the primers listed in **Table 1**. The PCR amplification conditions were as follows: an initial cycle at 95°C for 30 seconds, followed by 45 cycles at 95°C for 5 seconds and 60°C for 30 seconds, concluding with a final cycle at 95°C for 5 seconds, 60°C for 60 seconds, and 95°C for 15 seconds. The relative levels of target genes were calculated and normalized to GAPDH levels, with all experiments conducted in triplicate. mRNA expression levels were determined using the Livak method (2−ΔΔCT).

**Table 1:** Sequences of primers used in the study.

Genes	Primers	Sequences
*A. fumigatus*		
*Rho*	Sense Antisense	5′-GACTACGTCCACCACCTACAG-3′ 5′-GGCTAGCATCTCCAGTCAGAG-3′
*RasA*	Sense Antisense	5′-GATTCAGAGTCACTTCGTCG-3′ 5′-CTAAGACATCCAACAAAGCG-3′
*RacA*	Sense Antisense	5′-GGGACTGTGGGATACTGCTG-3′ 5′-GCTCAATCTCCGGGTACCAC-3′
*GAPDH1*	Sense Antisense	5′-GCCTCTTAAGGGTATCCTGACCTA-3′ 5′-TACCAGCTCACCAACTTCACGA-3′
Mouse		
IL-1β	Sense Antisense	5′-TGGACCTTCCAGGATGAGGACA-3′ 5′-GTTCATCTCGGAGCCTGTAGTG-3′
IL-6	Sense Antisense	5′-CTTGCAAGACTTCCATCCAC-3′ 5′-AGTGGTAGACAGGTCTGTTGC-3′
TNF-α	Sense Antisense	5′-CCTGTAGCCCACGTCGTAC-3′ 5′-GGGAGTAGACAAGGTACAACCC-3′
CCL-2	Sense Antisense	5′-CTCAGCCAGATGCAGTTAACG-3′ 5′-CAGACCTCTCTCTTGAGCTTGG-3′
CCL-7	Sense Antisense	5′-GCTGCTTTCAGCATCCAAGTG-3′ 5′-CCAGGGACACCGACTACTG-3′
CXCL-2	Sense Antisense	5′-GTTGACTTCAAGAACATCCAG-3′ 5′-CTTTCTCTTTGGTTCTTCCG-3′
CXCL-3	Sense Antisense	5′-ATCCCAACGGTGTCTGGATG-3′ 5′-GCAAGTAGATGCAATTATACCCGT-3′
MMP9	Sense Antisense	5′-GCCCTGGAACTCACACGACA-3′ 5′-TTGGAAACTCACACGCCAGAAG-3′
MMP13	Sense Antisense	5′-GATGGACCTTCTGGTCTTCT-3′ 5′-GCTCATGGGCAGCAACAATA-3′
GAPDH	Sense Antisense	5′-GGTTGTCTCCTGCGACTTCA-3′ 5′-TGGTCCAGCGTTTCTTACTCC-3′

### 2.10 Enzyme-linked immunosorbent assay

To evaluate the concentrations of TNF-α, IL-1β, and IL-6 in either the cell culture medium or the bronchoalveolar lavage fluid (BALF) obtained from mouse models of CSLI, cytokine levels were measured using ELISA kits according to the manufacturer’s instructions. The specific kits used were JL10484-96T for TNF-α, JL18442-96T for IL-1β, and JL20268-96T for IL-6, all provided by J&L Biological Industrial, Shanghai, China. Each sample was analyzed in duplicate.

### 2.11 Protein extraction and Western blotting

MH-S lung macrophages were stimulated with a 1% concentration of supernatant derived from Af293 conidia during the 12-hour germ tube elongation phase (Af293-12h) for durations of 6 and 12 hours. The harvested MH-S cells were washed with ice-cold PBS and subsequently lysed using RIPA lysis solution (KGB5303, KeyGEN BioTECH, China), which contained protease and phosphatase inhibitors. Following extraction, the protein content of each sample was quantified using the BCA protein assay kit (KGB2101, KeyGEN BioTECH, China). Cellular proteins were then separated by electrophoresis on 10% SDS-PAGE gels and transferred to polyvinylidene fluoride (PVDF) membranes (Durapore^®^ Millipore). The membranes were blocked in 5% skim milk in TBST for 1 hour at room temperature and then incubated with the primary antibody overnight at 4°C. The proteins of interest included AKT, JAK1, STAT1, JNK, ERK, as well as their phosphorylated forms: AKT, JAK1, STAT1, JNK (Thr183/Tyr185), and ERK (Thr202/Tyr204), along with GAPDH as a loading control. After washing the membranes with TBST, they were incubated with either horseradish peroxidase (HRP)-conjugated polyclonal goat anti-mouse or HRP-conjugated polyclonal goat anti-rabbit antibodies for 1 hour at room temperature, followed by three washes with TBST. Subsequently, the ECL reagent (Abbkine) was applied to the membranes, and luminescence imaging was performed using a Bio-Rad ChemiDoc imaging system. The density of each band was compared with the corresponding control band and normalized against GAPDH using densitometry with ImageJ software (version 2.3.0). Results are expressed as a percentage of the untreated control.

### 2.12 Metabolomic analysis of *Aspergillus fumigatus* supernatant

This study analyzed metabolites using an ACQUITY UPLC I-Class Plus ultra-high-performance liquid chromatography-tandem mass spectrometry system, coupled with a QE Plus high-resolution mass spectrometer and relevant databases including METLIN, Lipidmaps, ChEBI (38–41). Samples were collected from two groups: pre-germination (4-hour swelling phase) and post-germination (12-hour germ tube elongation phase), with six biological replicates per group, resulting in a total of 12 samples. A 1 mL sample was extracted using the solid-phase extraction (SPE) column, followed by the collection of 3 mL of methanol eluent. The sample underwent nitrogen blowdown using a nitrogen evaporator. After drying under nitrogen, 300 μL of pre-chilled methanol-water (V:V = 4:1, containing a mixed internal standard at 4 μg/mL) was added and vortexed for one minute. The mixture was subjected to ultrasonic treatment in an ice-water bath for 10 minutes and then allowed to stand overnight at -40°C. Subsequently, it was centrifuged for 10 minutes at 12,000 rpm and 4°C. A total of 150 μL of the supernatant was drawn using a syringe, filtered through a 0.22 μm organic phase syringe filter, and transferred to an LC injection vial. The samples were stored at -80°C until LC-MS analysis. Mass spectrometry was performed in both positive and negative ion modes. The raw LC-MS data underwent baseline filtering, peak identification, integration, retention time correction, peak alignment, and normalization, facilitated by Progenesis QI V2.3 software (Nonlinear Dynamics, Newcastle, UK). Principal Component Analysis (PCA) was employed to visualize the overall distribution among samples and to assess analytical stability throughout the process. Orthogonal Partial Least Squares Discriminant Analysis (OPLS-DA) and Partial Least Squares Discriminant Analysis (PLS-DA) were utilized to distinguish metabolite differences between the groups. Variable Importance Projection (VIP) values derived from the OPLS-DA model ranked each variable’s overall contribution to group discrimination. Bilateral Student’s t-tests were subsequently applied to validate the statistical significance of inter-group metabolite differences. Differential metabolites with VIP values exceeding 1.0 and p-values below 0.05 were selected and annotated for metabolic pathways via the Kyoto Encyclopedia of Genes and Genomes (KEGG) database (42), identifying the metabolic pathways in which these differential metabolites participated.

### 2.13 Statistical analysis

Statistical analysis was conducted using GraphPad Prism 10.0 (La Jolla, USA). Data are presented as mean ± standard deviation (SD) and were analyzed using the unpaired Student’s t-test for two groups or one-way analysis of variance (ANOVA) with Dunnett’s *post hoc* test for three or more groups, followed by the Bonferroni *post hoc* test. A *p*-value of less than 0.05 was considered indicative of significant differences.

## 3 Results

### 3.1 Morphological characteristics of Af293 conidial germination

**Figure 1:**
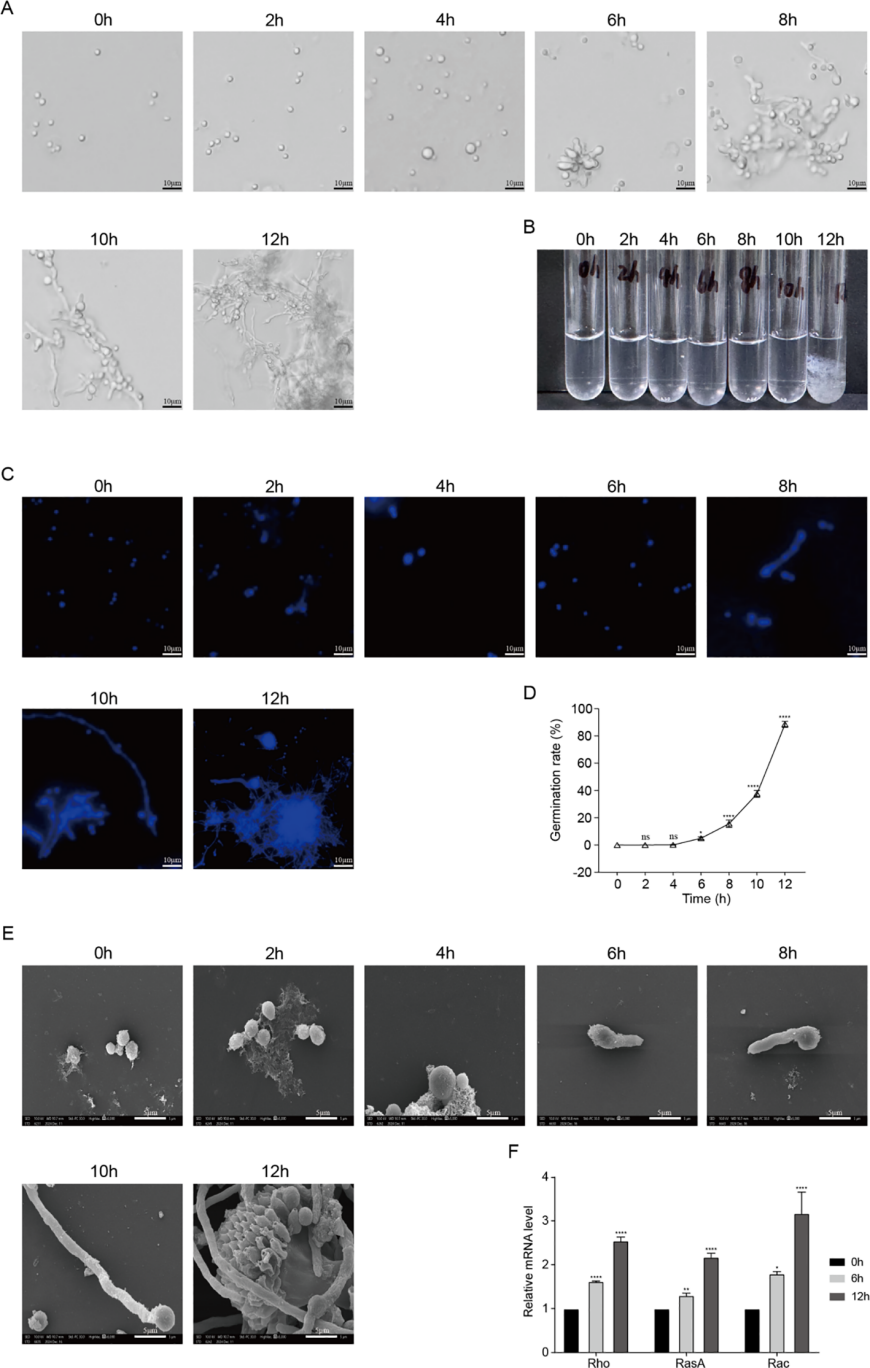
Morphological characteristics of conidial germination of *A*. *fumigatus* Af293. **(A)** Morphological observations of *A*. *fumigatus* at different germination times under an optical microscope (scale bar = 10 um); **(B)** Morphological observations of Af293 at different germination times in culture media; **(C)** DAPI staining of conidia with different morphologies (scale bar = 5 um); **(D)** Germination rates of Af293 conidia at various time points; **(E)** Morphological observations of Af293 at different germination times under a scanning electron microscope (scale bar = 5 um); **(F)** Relative mRNA levels of polar growth-related genes *Rho*, *RasA*, and *Rac* as determined by RT-qPCR. (*p < 0.05, **p < 0.01, ***p < 0.001, ****p < 0.001 in one-way ANOVA with Tukey’s multiple comparison).

To observe the morphological characteristics during the early germination process of Af293 conidia, we selected a culture duration of 0 to 12 hours, employing optical microscopy, DAPI staining, and scanning electron microscopy. The results indicated that the conidia began to swell significantly at 4 hours, with a small number initiating germination at 6 hours, and the majority completing germination after 12 hours, as observed both under the optical microscope and in test tubes (**Figures 1A, B**). Consequently, we propose that the conidia undergo a water absorption and swelling phase from 0 to 4 hours, a polar growth phase from 4 to 8 hours, and a germ tube growth phase from 8 to 12 hours. DAPI staining revealed that changes in nuclear division occurred during the water absorption and swelling phase, with an increase in the number of nuclei during the polar growth phase (**Figure 1C**). The germination rate also significantly increased between 6 and 12 hours (**Figure 1D**), which aligns with the results obtained from electron microscopy (**Figure 1E**). We selected three time points: pre-germination (0h), polar growth phase (6h), and germ tube growth phase (12h), and measured the mRNA expression levels of polar growth-related genes during the germination process using RT-qPCR. We found that, compared to 0h, the mRNA levels of *Rho*, *RasA*, and *Rac* were significantly elevated at 6h and 12h (**Figure 1F**). These results indicate that Af293 conidia exhibit distinct morphological characteristics at various germination stages and demonstrate a clear time-dependent relationship.

### 3.2 Overall sample differential analysis of conidial supernatant

**Figure 2:**
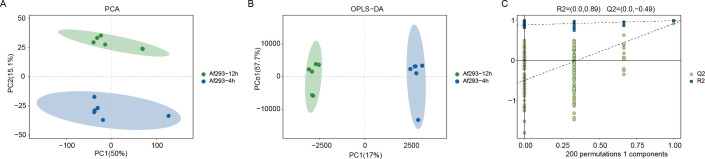
Overall metabolic differential analysis of conidial supernatant between pre-germination (Af293-4h) and post-germination (Af293-12h) groups. **(A)** Principal Component Analysis (PCA) plot; **(B)** Orthogonal Partial Least Squares Discriminant Analysis (OPLS-DA) plot; **(C)** Permutation validation plot.

Previous results have demonstrated significant morphological differences in the conidia of Af293 between the pre-germination and germination stages, indicating potential metabolic variations in the secreted metabolites during these phases. In our study, the 4-hour point (Af293-4h) corresponds to the pre-germination and early swelling phase, during which conidia have absorbed water, initiated isotropic growth, and begun transcriptional activation, yet have not produced a visible germ tube. In contrast, the 12-hour point (Af293-12h) represents the post-germination and early hyphal outgrowth phase, characterized by the establishment of polarized growth and a marked reprogramming of primary metabolism. Therefore, we employed mass spectrometry to analyze the metabolic differences in the conidial supernatant between the pre-germination (Af293-4h) and post-germination (Af293-12h) stages. The results of the Principal Component Analysis (PCA) revealed that the first principal component accounted for 50% of the variance, while the second principal component accounted for 15.1%. The relative distances among points within each sample group were notably small and tightly distributed, indicating a concentrated distribution; however, a significant trend of separation between the groups was observed (**Figure 2A**). To further investigate the differences in metabolites, we employed Orthogonal Partial Least Squares Discriminant Analysis (OPLS-DA) to eliminate irrelevant data variations, thereby enhancing the capture of differential metabolite information between the two groups. We observed significant differences between the two sample groups in the OPLS-DA score plot (**Figure 2B**). Additionally, we performed a permutation test with 200 iterations on the model, randomly permuting the variables of the previously defined classification Y matrix (e.g., 0 or 1) n times (n=200) to establish corresponding OPLS-DA models and obtain the R² and Q² values of the random models. As illustrated in **Figure 2C**, R² = 0.89 exceeds Q² = -0.49, and the Q² regression line intercept with the Y-axis is less than 0, indicating that the model possesses reasonable predictive capability. The model demonstrates robust explanatory power for the samples, accurately reflecting the information contained within them (**Figure 2C**).

### 3.3 Differential metabolite analysis of conidial supernatant

**Figure 3:**
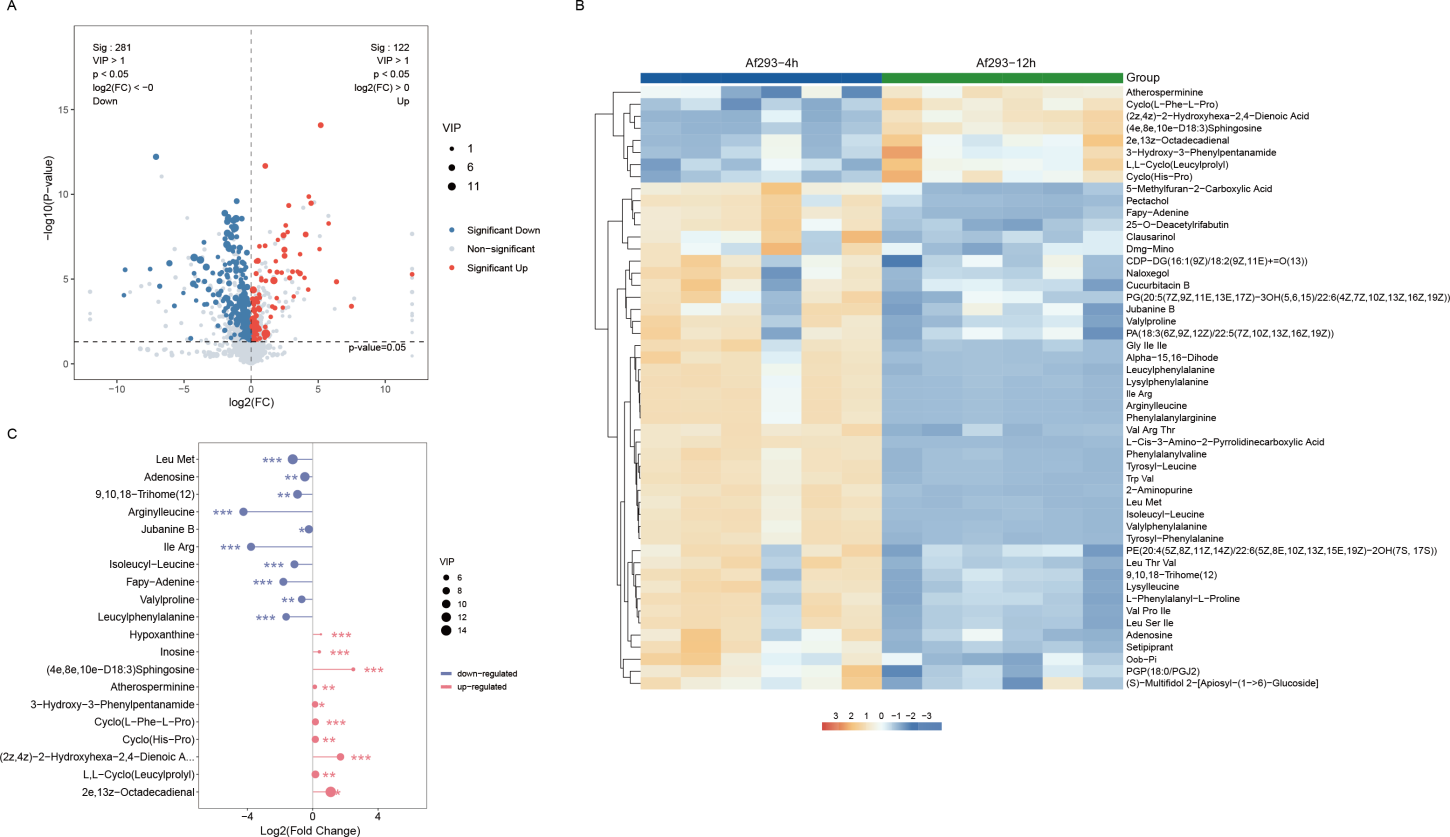
Analysis of differential metabolites between pre-germination (Af293-4h) and germination (Af293-12h) groups. **(A)** Volcano plot; **(B)** VIP Top 50 clustering heatmap; **(C)** Upregulated and downregulated differential metabolites VIP Top 10 lollipop chart.

Based on the OPLS-DA model, the variable importance in projection (VIP) values for each metabolite were obtained, with a general consensus that metabolites with VIP > 1 are statistically significant. The results from the volcano plot indicate that in the Af293-12h group, 40 metabolites are upregulated and 101 metabolites are downregulated compared to the Af293-4h group, adhering to the screening criteria of *p*-value < 0.05, fold change (FC) > 2 or FC < 0.5, and VIP > 1 (**Figure 3A**). These metabolites primarily consist of organic acids and their derivatives, lipids and lipid-like molecules, as well as organic heterocyclic compounds. We performed hierarchical clustering on all significantly different metabolites based on the expression levels of the top 50 VIP values; the hierarchical clustering heatmap clearly illustrates significant differences in metabolite abundance between the two sample groups. Compared to the Af293-4h group, the abundances of Atherosperminine, Cyclo(L-Phe-L-Pro), (2z,4z)-2-Hydroxyhexa-2,4-Dienoic Acid, (4e,8e,10e)-D18:3 Sphingosine, 2e,13z-Octadecadienal, 3-Hydroxy-3-Phenylpentanamide, L,L-Cyclo(Leucylprolyl), and Cyclo(His-Pro) significantly increased in the Af293-12h group. In contrast, the abundances of 5-Methylfuran-2-Carboxylic Acid, Pectachol, Fapy-Adenine, and 42 other metabolites markedly decreased in the Af293-12h group (**Figure 3B**). We presented the top ten differential metabolites with the highest VIP values in **Figure 3C** and **Table 2**. Compared to the Af293-4h group, the metabolites that exhibited a decrease in the Af293-12h group include Leu Met, adenosine, 9,10,18-Trihome (12), arginylleucine, jubanine B, Ile Arg, isoleucyl-leucine, fapy-adenine, valylproline, and leucylphenylalanine. In addition to the eight significantly elevated metabolites indicated in the heatmap, both Hypoxanthine and Inosine also demonstrated increased levels in the Af293-12h group.

**Table 2:** Top 10 Metabolites with up-regulated and down-regulated differences.

Metabolites	Retention time (min)	Formula	Mass-to-charge ratio (m/z)
Leu Met	3.85076666666667	C11H22N2O3S	263.139784717201
Adenosine	1.17188333333333	C10H13N5O4	312.09551943557
9,10,18-Trihome (12)	7.12376666666667	C18H34O5	329.233850316604
Arginylleucine	1.31453333333333	C12H25N5O3	288.203028787236
Jubanine B	4.64061666666667	C43H47N5O6	730.360110195805
lle Arg	1.15586666666667	C12H25N5O3	288.203092939169
Isoleucyl-Leucine	3.97401666666667	C12H24N2O3	245.186438320647
Fapy-Adenine	0.751833333333333	C5H7N5O	136.062203943373
Valylproline	1.81563333333333	C10H18N2O3	215.13936196682
Leucylphenylalanine	4.50646666666667	C15H22N2O3	279.170572830627
Hypoxanthine	1.30138333333333	C5H4N4O	137.046106610468
Inosine	1.28685	C10H12N4O5	269.088312408884
(4e, 8e, 10e-D18:3) Sphingosine	10.6695333333333	C18H33NO2	296.258778971969
Atherosperminine	4.3397	C20H23NO2	327.206689275614
3-Hydroxy-3-Phenylpentanamide	4.61111666666667	C11H15NO2	211.144513581141
Cyclo(L-Phe-L-Pro)	4.94325	C14H16N2O2	245.128782644162
Cyclo(His-Pro)	0.7632	C11H14N4O2	235.119638001593
(2z, 4z)-2-Hydroxyhexa-2,4-Dienoic Acid	1.97526666666667	C6H8O3	111.04465425608
L, L-Cyclo(Leucylprolyl)	4.74638333333333	C11H18N2O2	211.144443254875
2e,13z-Octadecadienal	12.6619	C18H32O	282.279423603985

### 3.4 KEGG pathway enrichment analysis of differential metabolites

**Figure 4:**
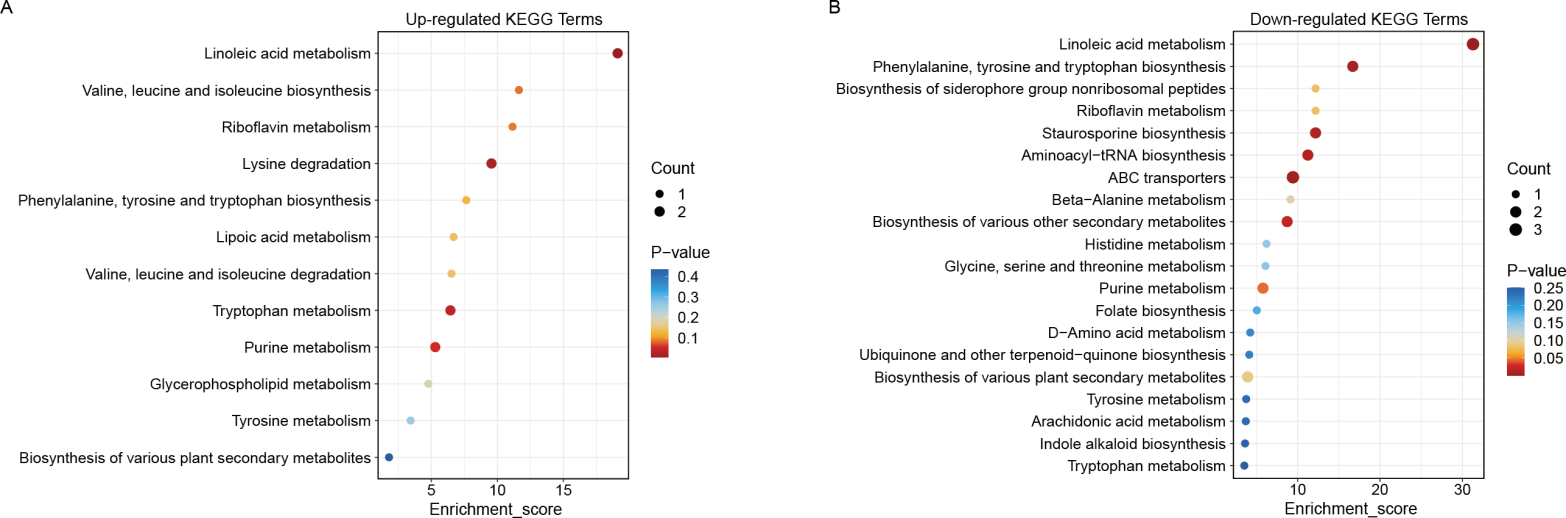
Enriched KEGG pathways in Af293-12h vs Af293-4h. **(A)** Upregulated metabolic pathways; **(B)** Downregulated metabolic pathways.

KEGG enrichment analysis was performed to explore the relationships between the differential metabolites and metabolic pathways. As illustrated in **Figure 4**, the top 20 pathways with the lowest *p*-values during the germination period (Af293-12h), in comparison to the water absorption swelling period (Af293-4h), are presented. The upregulated pathways that were significantly enriched include linoleic acid metabolism, lysine degradation, and tryptophan metabolism (*p* < 0.05) (**Figure 4A**). Conversely, the downregulated pathways are primarily enriched in linoleic acid metabolism, ABC transporters, the biosynthesis of phenylalanine, tyrosine, and tryptophan, astaxanthin biosynthesis, aminoacyl-tRNA biosynthesis, the biosynthesis of various secondary metabolites, and purine metabolism (*p* < 0.05) (**Figure 4B**).

### 3.5 Inflammatory response in MH-S cells induced by conidial supernatant

**Figure 5:**
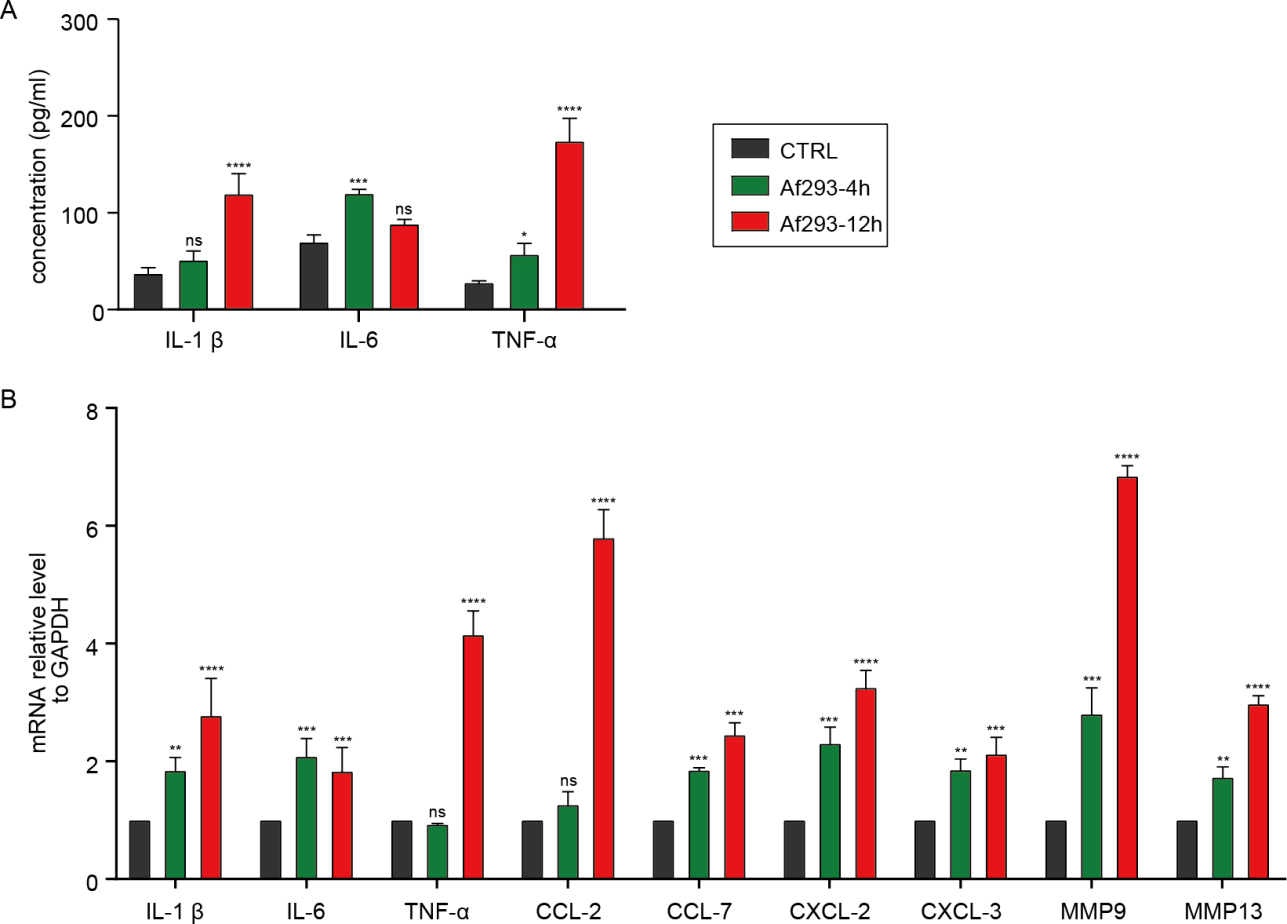
Levels of inflammatory factors in MH-S cells treated with supernatant from Af293 at 4 hours (Af293-4h) and 12 hours (Af293-12h). **(A)** The concentration of inflammatory factors in the cell supernatant was detected using ELISA; **(B)** The mRNA levels of inflammatory factors within the cells were assessed through RT-QPCR. (*p<0.05, **p<0.01, ***p<0.001, ****p<0.001 in one-way ANOVA with Tukey’s multiple comparison).

Although *A. fumigatus* conidia can elicit an inflammatory response in macrophages, the effects of conidial supernatant over varying time periods on macrophage inflammatory response remain unclear. We treated MH-S cells with supernatant from Af293 at 4 hours and 12 hours, subsequently assessing the expression of inflammatory factors using ELISA and RT-qPCR. The ELISA results indicated that both the Af293-4h and Af293-12h groups induced the expression of inflammatory factors IL-1β and TNF-α, with levels in the Af293-12h group significantly higher than those in the Af293-4h group (**Figure 5A**). The RT-qPCR results revealed that levels of IL factors, including IL-1β and TNF-α, chemokines such as CCL-2, CCL-7, CXCL-2, and CXCL-3, as well as metalloproteinases including MMP-9 and MMP-13, were all elevated in the Af293-12h group compared to the Af293-4h group (**Figure 5B**). This indicates that the supernatant from germinated Af293 can induce a stronger inflammatory response.

### 3.6 Conidial supernatant induced inflammation through the activation of JAK-STAT and MAPK pathways

**Figure 6:**
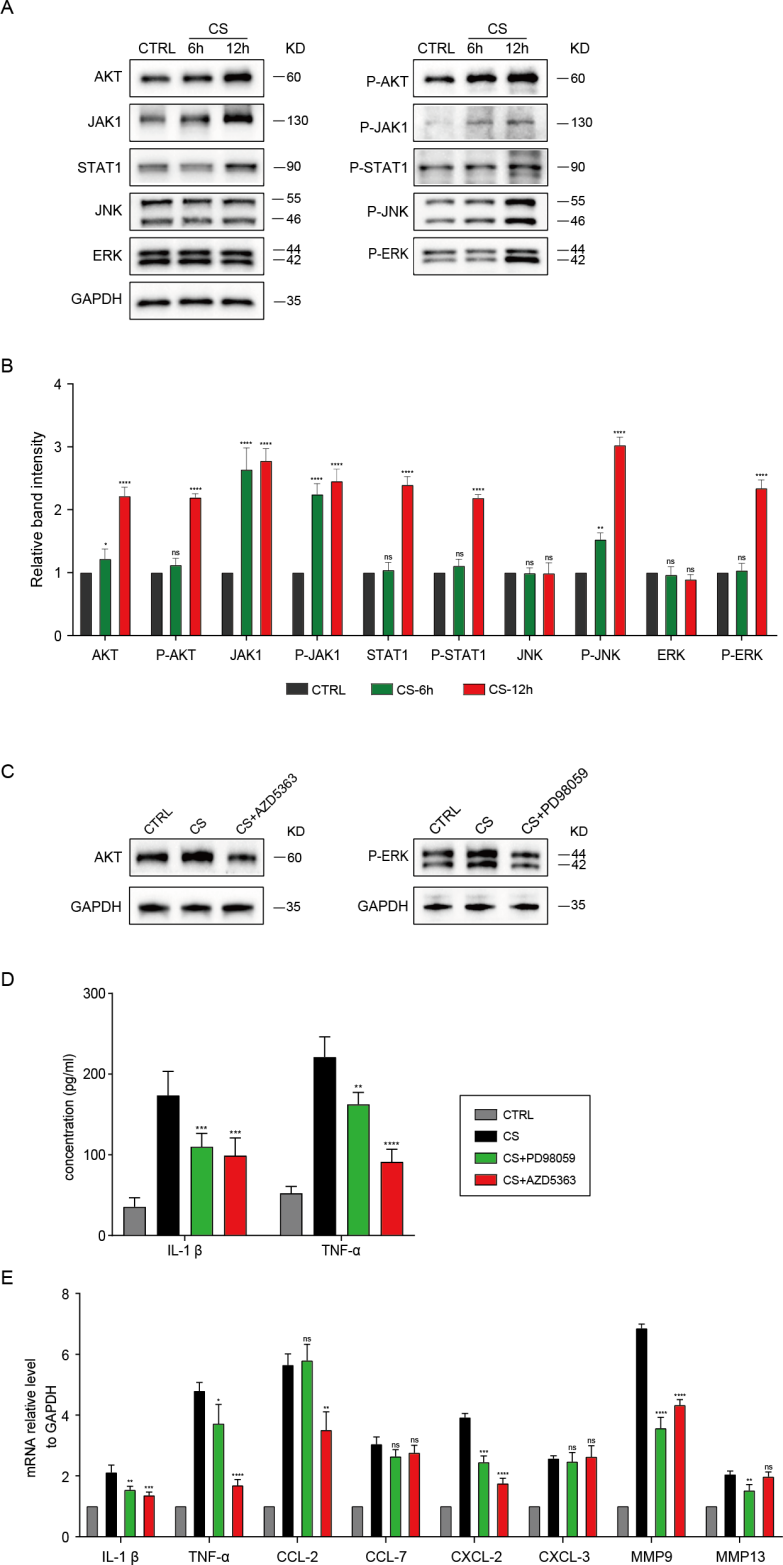
The total protein and phosphorylation levels of the JAK-STAT and MAPK pathways in MH-S cells treated with Conidial Supernatant (CS) were assessed at 6 and 12 hours. **(A)** The total levels of proteins and phosphorylated proteins involved in the JAK-STAT and MAPK pathways were assessed using Western blotting. **(B)** The relative band intensity from **Figure 6A** was quantified. **(C)** The inhibitory effects of the AKT inhibitor (AZD5363, 50uM) on AKT and the phosphorylated-ERK inhibitor (PD98059, 50uM) on phosphorylated-ERK were assessed using Western blotting. **(D)** The concentration of inflammatory factors in treated cells with inhibitors AZD5363 or PD98059 were measured using ELISA. E: The mRNA levels of inflammatory factors in cells treated with the inhibitors AZD5363 or PD98059 were measured using RT-qPCR. (*p < 0.05, **p < 0.01, ***p < 0.001, ****p < 0.001 in one-way ANOVA with Tukey’s multiple comparison).

Infection with *A. fumigatus* can activate various inflammatory pathways, including the JAK-STAT (43, 44) and MAPK (20, 45) pathways, which have not been previously reported to be induced by the conidial supernatant. To investigate this, we stimulated MH-S cells with the Conidial Supernatant (CS) from Af293-12h, as it is known to induce a more robust inflammatory response. We then assessed the overall protein levels and phosphorylation states of key proteins involved in inflammatory signaling pathways, including JAK1, STAT1, AKT, JNK, and ERK, using Western blot (WB) analysis. The results indicated that the phosphorylation levels of these proteins were significantly elevated after 12 hours of CS treatment, suggesting the activation of these pathways (**Figures 6A, B**). To elucidate the signaling pathways activated by CS that lead to inflammatory responses, we employed the AKT inhibitor (AZD5363, 50uM) and the phosphorylated-ERK inhibitor (PD98059, 50uM) to inhibit the relevant pathway proteins and evaluate the alterations in downstream inflammatory factors. **Figure 6C** shows that AZD5363 and PD98059 effectively inhibited the expression of AKT and phosphorylated-ERK, respectively. As illustrated in **Figure 6D**, both AZD5363 and PD98059 significantly reduced the elevated concentrations of the inflammatory factors IL-1β and TNF-α induced by CS. The RT-qPCR results (**Figure 6E**) indicated that AZD5363 significantly decreased the mRNA levels of the inflammatory factors IL-1β, TNF-α, CCL-2, CXCL-2, and MMP9, while PD98059 resulted in a reduction of the mRNA levels of IL-1β, CXCL-2, MMP9, and MMP13. These findings imply that CS-12h promotes the phosphorylation of multiple inflammatory-related proteins, thereby stimulating MH-S cells to produce elevated levels of inflammatory factors and response.

### 3.7 Conidial supernatant induced lung inflammation in mice

**Figure 7:**
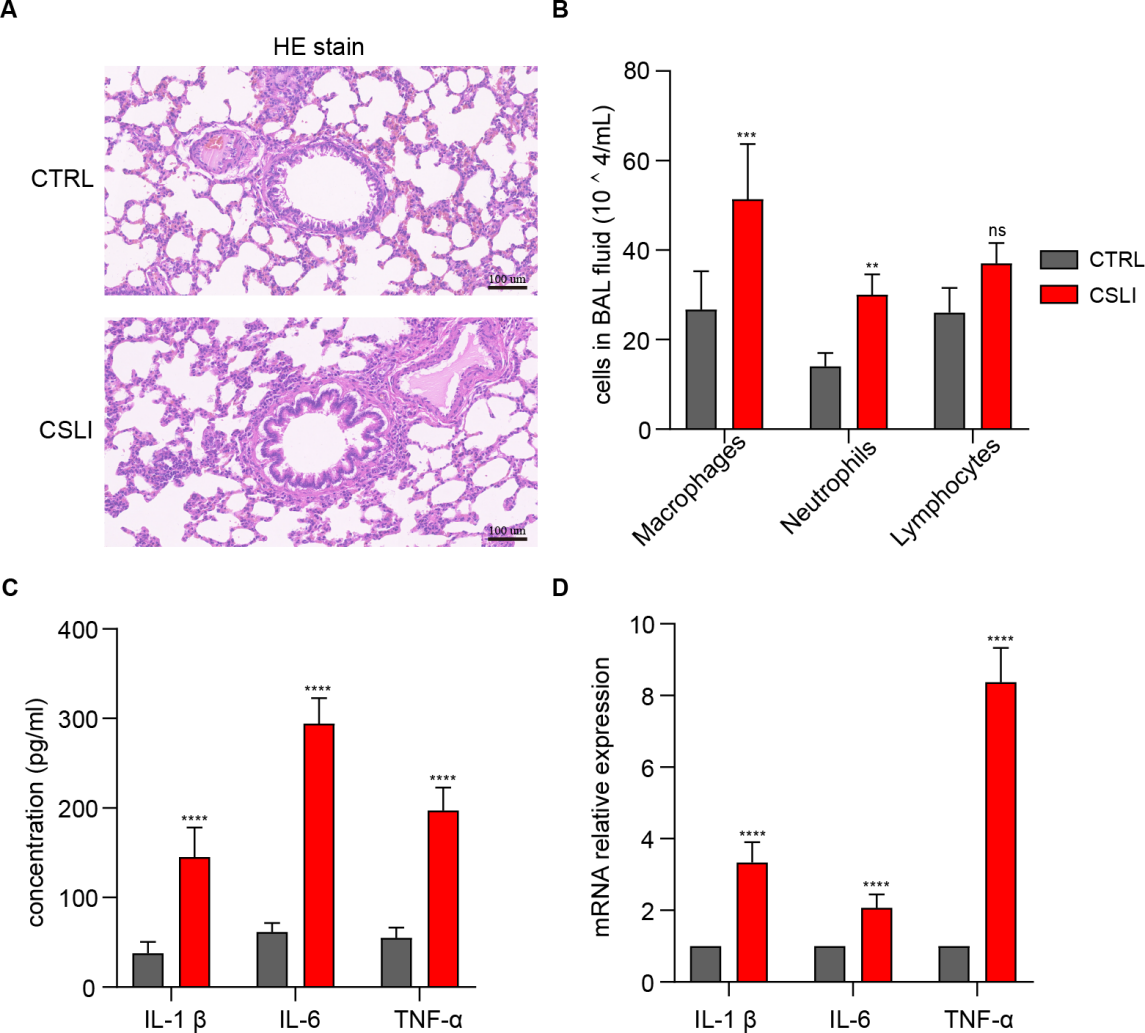
Long-term nasal instillation of conidial supernatant induced lung inflammation (CSLI) in mice. **(A)** HE staining shows pathological sections of lung tissue from CTRL (Control) and CSLI mice. **(B)** Giemsa staining reveals the presence of macrophages, neutrophils, and lymphocytes in the bronchoalveolar lavage fluid. **(C)** ELISA demonstrates the concentration of inflammatory factors TNF-α, IL-1β, and IL-6 in the bronchoalveolar lavage fluid. **(D)** RT-qPCR indicates the mRNA level of inflammatory factors in the lung tissue of mice. (*p < 0.05, **p < 0.01, ***p < 0.001, ****p < 0.001 in a two-tailed unpaired t-test).

Conidial supernatant has the capacity to stimulate inflammation in MH-S cells; however, its potential to induce lung inflammation *in vivo* remains unclear. To address this issue, we established a mouse pneumonia model by continuously administering conidial supernatant via intranasal instillation for one month, referred to as the CLSI group. The control group received intranasal instillation of PBS. Hematoxylin and eosin (HE) staining revealed inflammatory cell infiltration and pulmonary emphysema in the lung tissue of mice in the CLSI group, indicating damage to lung tissue caused by conidial supernatant (**Figure 7A**). Cytological analysis using Giemsa staining demonstrated a marked increase in macrophages and neutrophils, along with a slight but statistically non-significant increase in lymphocytes in the bronchoalveolar lavage fluid (BALF) of mice in the CLSI group compared to the control group (**Figure 7B**). At the molecular level, ELISA results showed significantly elevated levels of pro-inflammatory cytokines (TNF-α, IL-1β, and IL-6) in the BALF of mice in the CLSI group (**Figure 7C**). This finding was corroborated by RT-qPCR analysis of inflammatory markers in lung tissue (**Figure 7D**). In summary, we have established a mouse model of chronic pneumonia induced by conidial supernatant, which provides a reliable platform for the in-depth investigation of host interactions with Af293 conidial supernatant.

## 4 Discussion

This study systematically elucidates, for the first time, the dynamic relationship between the conidia germination process of *A. fumigatus* and its pathogenicity. As conidia germinate, *A. fumigatus* undergoes significant metabolic reprogramming, accompanied by toxin accumulation. Non-targeted metabolomics analysis reveals an increased secretion of organic acids and derivatives, lipids and lipid-like molecules, phenolic compounds, phenylpropanoids, polyketides, as well as alkaloids and their derivatives following conidia germination. Moreover, the metabolic characteristics of its secretome and its capacity to induce host inflammatory response are dynamic, markedly intensifying over time. Compared to pre-germination supernatant, those from the post-germination supernatant induce significantly stronger inflammatory response *in vitro*. These secondary metabolites activate host inflammatory response *in vitro* via the JAK/STAT/AKT and MAPK pathways. Thus, studying the inflammatory response mediated by conidial supernatant serves as a crucial complement to the mechanisms of conidia infection. This approach provides significant insights into the comprehensive understanding of the pathogenic process of *A. fumigatus*.

Our morphological observations indicate that the conidia transition from a dormant to an active germination state between 4 and 12 hours. These two time points encapsulate the primary differences in metabolic states before and after germination, facilitating the identification of key metabolites and pathway alterations associated with the initiation of germination. Consistent with this observation, mass spectrometry analysis revealed significant differences in the metabolites present in the supernatant at these two time points. Overall, the tryptophan metabolic pathway in *A. fumigatus* is markedly upregulated. Existing literature confirms that *A. fumigatus* produces multiple tryptophan-containing toxins, including fumigaclavine C, fumiquinazoline C, fumitremorgin, gliotoxin, and hexadehydroastechrome (46). The synthesis of these toxins depends on tryptophan, which provides essential structural components (47). Given that humans and other mammals cannot synthesize tryptophan, the ability of *A. fumigatus* to autonomously produce this amino acid enables it to sustain growth and continuously synthesize toxins in nutritionally limited environments, such as the lungs of hosts. This metabolic advantage enables the fungus to dominate during infection without relying on tryptophan supplied by the host (48). On the other hand, our results indicate a significantly upregulated linoleic acid metabolic pathway during the germination of *A. fumigatus*. This finding is corroborated by other studies that demonstrate *A. fumigatus* metabolizes linoleic acid through specific oxidase systems, such as lipoxygenases (49). Furthermore, studies have confirmed that 5,8-diHODE, an endogenously produced and secreted dihydroxyoxylipin, regulates the differentiation process of filamentous fungi, including lateral branching in pathogenic *A. fumigatus* and *Aspergillus flavus* (50). These findings suggest that germinating conidia do not passively release substances; rather, they actively undergo ‘metabolic reprogramming,’ potentially altering the nature of their interactions with the host immune system. However, we must acknowledge that selecting only two time points (4 hours and 12 hours) for metabolite detection is insufficient. Consequently, future studies will incorporate additional time points for metabolite detection during conidia growth.

Functional assays confirmed the biological consequences of these metabolic alterations. We first demonstrated that the conidial supernatant of Af293-12h induced a greater release of inflammatory cytokines in cellular models compared to the conidial supernatant of Af293-4h. We hypothesize that the enhanced pro-inflammatory capacity of the CS-12h may arise from the accumulation of certain metabolites, such as diketopiperazine compounds, including L, L-Cyclo (Leucylprolyl), Cyclo (L-Phe-L-Pro), and Cyclo (His-Pro). Notably, Cyclo(L-Phe-L-Pro) has been previously reported (51). These can be regarded as core skeletons or direct biosynthetic precursors of gliotoxin and its related mycotoxins. The elevated metabolite (2Z,4Z)-2-hydroxyhexa-2,4-dienoic acid may serve as a key intermediate or degradation product in the biosynthetic pathway of polyketides, based on its chemical structure. Certain metabolites, such as hypoxanthine and inosine, which exhibit marked increases, may play a role in the adaptive survival and sustained growth of *A. fumigatus*. For *A. fumigatus*, sphingosine is essential for maintaining cell wall integrity and toxicity (52, 53). Sphingolipid signaling is critical for the formation of fungal biofilms, which are vital for persistent infection and drug resistance (54, 55). Among these differential metabolites, it is necessary to conduct further experiments to confirm which specific metabolites are responsible for the inflammatory response.

Inflammation triggered by *A. fumigatus* conidia infection is characterized as a “biphasic” process. The initial phase of inflammation is primarily initiated by the physical interaction between pathogen-associated molecular patterns (PAMPs) present on the conidia surface and host pattern recognition receptors, such as Dectin-1 and Toll-like receptors (TLRs), leading to rapid and intense signaling (19, 22). The later phase, which coincides with conidia germination and hyphal formation, is marked by the continuous secretion of effectors, including toxins and proteases, which persistently intensify and amplify the inflammatory response, potentially resulting in more severe damage to host cells. Previous studies have indicated that *A. fumigatus* conidia can stimulate host cells and activate the phosphorylation of the PI3K/AKT, JAK/STAT, and MAPK pathways (44, 56, 57). Our findings reveal that the conidial supernatant stimulation leads to elevated levels of inflammatory protein phosphorylation in cells, including PI3K/AKT, JAK/STAT, and MAPK pathways. Furthermore, the inhibition of ERK and AKT resulted in a significant decrease in the inflammatory factors IL-1β, TNF-α, and CXCL-2. A study conducted by Xiao Cui et al. demonstrated that *A. fumigatus* conidia can activate the MAPK signaling pathway in BEAS-2B epithelial cells, resulting in the induction of cytokine production (58). Notably, the application of a JNK inhibitor reversed the secretion of inflammatory factors induced by *A. fumigatus* in BEAS-2B cells, whereas inhibitors of p38 MAPK and ERK did not influence these processes. This discrepancy may stem from the differences in MAPK pathways between epithelial cells and macrophages. In a separate study (59), exposure of BEAS-2B cells to *A. fumigatus* conidia activated the AKT/mTOR pathway, leading to cellular inflammation and apoptosis. Furthermore, *A. fumigatus*s conidia stimulated the release of cytokines from dendritic cells, promoting Th17 responses, including IL-1β, IL-6, and IL-23, via the JAK/STAT signaling pathway (44). These studies indicate that conidia or conidial supernatant can promote inflammatory responses by activating various signaling pathways. *Aspergillus*-mediated allergic airway inflammation was confirmed through repeated intranasal exposure to *A. fumigatus* conidia (A1160) in mouse model (60). Another study revealed that immunocompetent mice subjected to *A. fumigatus* conidia (AFsp group) exhibited a pronounced inflammatory response, characterized by an increase in the number of bronchoalveolar lavage fluid (BALF) macrophages and elevated expression of inflammatory factors (IL-1β, CXCL-2, CXCL-1, GM-CSF, IL-6), which are associated with lung injury (8). These findings align with our established pneumonia model in immunocompetent mice, developed through nasal instillation of *A. fumigatus* conidial supernatant over one month. Consequently, prolonged nasal exposure to conidial supernatant or conidia induces similar pulmonary inflammation in the mice.

However, this study has several limitations. First, the supernatant represents a complex mixture of components. Although we have identified key metabolites through correlation analysis, further research is necessary for metabolite quantification and to ascertain which metabolites play significant roles in the inflammatory response. Second, it is essential to validate pneumonia induced by the supernatant of *A. fumigatus* in a larger cohort of mice. The duration and frequency of exposure to supernatant stimulation must be repeated and confirmed, and additional pathological analyses of pneumonia in mice should be conducted. Third, although the conidial supernatant cannot form colonies on PDA plates, the possibility of residual protein components that may induce inflammatory responses cannot be excluded. Therefore, we need to assess the purity of the supernatant through controls, including proteinase K treatment or heat inactivation, and we will subsequently reevaluate the inflammatory effects in future studies.

## 5 Conclusion

Our research indicates significant differences in the metabolites of *A. fumigatus* conidial supernatant between the pre-germination and post-germination stages. The conidial supernatant can induce a pronounced inflammatory response in macrophages, mediated by the activation of the JAK/STAT/MAPK pathways. Long-term exposure to spore supernatant in mice can result in pneumonia and tissue damage. Therefore, the development of preventive or intervention strategies targeting these specific metabolites or the inflammatory pathways they activate presents significant potential.

## Data Availability

The data presented in the study are deposited in the OMIX repository, China National Center for Bioinformation/Beijing Institute of Genomics, Chinese Academy of Sciences, accession number OMIX010709.
